# Influence of Exercise on Oxygen Consumption, Pulmonary Ventilation, and Blood Gas Analyses in Individuals with Chronic Diseases

**DOI:** 10.3390/life15081255

**Published:** 2025-08-07

**Authors:** Mallikarjuna Korivi, Mohan Krishna Ghanta, Poojith Nuthalapati, Nagabhishek Sirpu Natesh, Jingwei Tang, LVKS Bhaskar

**Affiliations:** 1 College of Physical Education and Health Sciences, Zhejiang Normal University, Jinhua 321004, China; mallik.k5@gmail.com; 2 Department of Pharmacology, MVJ Medical College and Research Hospital, Hoskote, Bengaluru 562114, Karnataka, India; gmkchowdary81@yahoo.co.in; 3 Department of Neurology, Massachusetts General Hospital, Harvard Medical School, Boston, MA 02114, USA; dr.poojithnuthalapati@gmail.com; 4 Department of Veterinary Medicine and Surgery, University of Missouri, Columbia, MO 65201, USA; nagabhishek.natesh@gmail.com; 5 Roy Blunt NextGen Precision Health Institute, University of Missouri, Columbia, MO 65211, USA; 6 Qixin College, Ningbo Tech University, Ningbo 315100, China; 7 Department of Zoology, Guru Ghasidas Vishwavidyalaya, Bilaspur 495009, Chhattisgarh, India

**Keywords:** oxygen consumption, pulmonary ventilation, blood gases, physical activity, metabolic diseases

## Abstract

The increasing prevalence of chronic metabolic diseases poses a significant challenge in the modern world, impacting healthcare systems and individual life expectancy. The World Health Organization (WHO) recommends that older adults (65+ years) engage in 150–300 min of moderate-intensity or 75–150 min of vigorous-intensity physical activity, alongside muscle-strengthening and balance-training exercises at least twice a week. However, nearly one-third of the adult population (31%) is physically inactive, which increases the risk of developing obesity, type 2 diabetes, cardiovascular diseases, hypertension, and psychological issues. Physical activity in the form of aerobic exercise, resistance training, or a combination of both is effective in preventing and managing these metabolic diseases. In this review, we explored the effects of exercise training, especially on respiratory and pulmonary factors, including oxygen consumption, pulmonary ventilation, and blood gas analyses among adults. During exercise, oxygen consumption can increase up to 15-fold (from a resting rate of ~250 mL/min) to meet heightened metabolic demands, enhancing tidal volume and pulmonary efficiency. During exercise, the increased energy demand of skeletal muscle leads to increases in tidal volume and pulmonary function, while blood gases play a key role in maintaining the pH of the blood. In this review, we explored the influence of age, body composition (BMI and obesity), lifestyle factors (smoking and alcohol use), and comorbidities (diabetes, hypertension, neurodegenerative disorders) in the modulation of these physiological responses. We underscored exercise as a potent non-pharmacological intervention for improving cardiopulmonary health and mitigating the progression of metabolic diseases in aging populations.

## 1. Introduction

According to the World Health Organization (WHO), physical activity is defined as any bodily movement produced by skeletal muscles that requires energy expenditure. For the first time, the WHO guidelines on “physical activity and sedentary behavior” recommend that older adults (above 65 years) should engage in a minimum of 150–300 min of moderate-intensity aerobic activity or at least 75–150 min of vigorous-intensity aerobic activity per week [1,2]. Alternatively, an equivalent combination of both moderate- and vigorous-intensity activity throughout the week is recommended [1,2]. A large proportion of adults (~31%) and adolescents (~80%) do not meet the WHO recommended guidelines on physical activity, thereby increasing the risk of developing chronic metabolic disorders [3]. A lack of physical activity is one of the leading risk factors for developing metabolic diseases, including obesity, type 2 diabetes (T2D), cardiovascular diseases (CVDs), hypertension, and disease-associated mortality. People with insufficient physical activity have a 20–30% greater risk of death compared to people with sufficient physical activity [3,4]. Physical activity in the form of aerobic exercise, resistance training, yoga, or Tai chi is known to offer several health benefits for all age groups of people. These benefits include reduced risk of chronic metabolic diseases, improved mental health, and enhanced overall well-being [4,5,6,7,8].

Owing to the increasing incidence of metabolic diseases among individuals with physical inactivity, greater attention has been given to workplace wellness and disease-prevention programs to improve employee health and reduce their healthcare costs. A previous study has shown that medical costs decreased by USD 1.585 for every USD 1 invested in a workplace wellness program [9]. Moreover, factors associated with health risk are linked with enhanced productivity at work [10,11]. Irregularities in physical activity or sedentary behavior can lead to various metabolic disorders like obesity, hypertension, body mass index (BMI) abnormalities, hyperglycemia, or metabolic syndrome. Previous research has demonstrated that ergometry performance has declined in individuals with normal weight and obesity [12], With every single type of physical work, oxygen (O_2_) intake is comparatively substantial, which is indicative of the high metabolic expenditure involved in exercising heavy limbs [12,13]. Comprehensive obesity rehabilitation (COR) programs, including cardiac rehabilitation, diet measures, nutrition counseling, and health education on weight gain, are typically associated with complications, and the quantity and intensity of exercise should be optimized in a personalized approach. This type of tailored exercise is used for patients with morbid obesity (high BMI) [14]. According to the WHO classification, BMI is defined as low when it is <18.5 kg/m^2^ (underweight), a normal BMI is between 18.5 and 24.9 kg/m^2^ (normal weight), a high BMI is between ≥25 and 29.9 kg/m^2^ (overweight), and a higher BMI is ≥ 30 (obesity) [15,16].

Moderate-intensity continuous training (MICT) is a popular training approach that offers several health and fitness benefits among individuals with metabolic and chronic diseases [8,17]; MICT is widely recommended to manage several diseases, such as obesity, diabetes, hypertension, and CVDs due to its accessibility and lower risk of injury compared to high-intensity training [17,18,19]. High-intensity interval training (HIIT) that entails short, intense workouts (less than 45 s) with a maximum heart rate of 85–95% interspersed with rest or lower-intensity active recuperation intervals is a useful method for achieving cardiorespiratory health [20]. As a time-efficient training method, HIIT induces cardiovascular and skeletal muscle adaptations and maximizes the advantages of exercise in health and disease conditions [21,22]. However, greater control of training variables is crucial when prescribing HIIT to lower the blood pressure in individuals with hypertension [23]. Exercise necessitates gas exchange, which involves the interplay of the circulatory, muscular, and ventilatory systems. The entire ability to exercise may be characterized by two parameters: activity time to exhaustion and maximal oxygen uptake [24].

### Exercise and Pathophysiological Conditions

Physical activity or exercise significantly influences the physiology of adults, and the effects depend on their health status, fitness levels, or any other pathological condition. This interplay between exercise and pathophysiological conditions is a complex area of study. Exercise can strengthen respiratory muscles and improve oxygen utilization, but it may cause negative effects if exercise is not tailored [25,26]. Pulmonary rehabilitation has been shown to enhance exercise tolerance and increase health-related quality of life. Pulmonary rehabilitation is widely considered a non-pharmacological treatment in patients with chronic obstructive pulmonary disease (COPD) [27]. One of the major causes of death in the United States and around the world is COPD. The exacerbation of COPD is defined as an acute worsening of respiratory symptoms beyond one’s ability to adapt normally, requiring additional treatment, and carries the risk of adverse health events that could affect the patient in the future [28]. Peak exercise oxygen uptake (VO_2_) is a common way to quantify cardiorespiratory fitness (CRF), which is an indicator of oxygen transport and utility integration between the cardiovascular system and tissues [29]. Another type of exercise is cardiac rehabilitation, which is used for heart failure patients and generally consists of 3–5 sessions/week that include walking, bicycling on an ergometer, and 40–60 min of calisthenics for 3 months. Parkinson’s disease, a neuromotor disorder, is one example of a neurodegenerative disease that causes respiratory abnormalities. These respiratory disorders typically combine restrictive as well as obstructive features, with limited lung capacity and greater effort for breathing [30,31]. In such cases, inspiratory muscle inadequacy and thoracic rigidity, as well as hyperkyphosis-like deformity, impede the expansion of the lungs and increase the risk of developing microatelectasis regions [32]. Furthermore, the condition results in brain stem lesions that induce a parasympathetic-predominant vegetative state. Patients who have elevated parasympathetic activity are more likely to experience upper airway obstruction in the glottis [33].

Several physiological circumstances, including vigorous exercise at sea level, fast climb to altitude, and a variety of cardiorespiratory disorders that result in a ventilation–perfusion gap, lead to arterial hypoxemia [34,35]. Exercise capacity is reduced in these scenarios, and symptoms like breathlessness emerge as a result of hypoxia-related alterations in metabolism and cardiorespiratory complications. The severity of manifestations is frequently correlated to the level of oxygen in the arterial system [36]. In many cases, acute oxygen treatment can cure the disturbances and manifestations caused by hypoxia; nevertheless, its general application is limited by logistic challenges as well as the adverse consequences of chronic oxygen therapy [37]. However, logistical impediments (e.g., availability and transportation of oxygen tanks) and the deleterious effects of chronic oxygen supplementation [38] limit its widespread application. The application of hypoxia conditioning as a weight reduction and health-promoting therapy for individuals with obesity has garnered more attention in recent years [39,40]. Significant changes in body weight, fat mass, blood sugar, cardiovascular fitness, and endurance, have been observed with hypoxia conditioning utilizing a fraction of inspired oxygen (FiO_2_) of 13–16.5% for one to two months, when compared to normoxic constant-load programs, such as 60–90 min walking/running/cycling, or cross-training with 60–70% maximum oxygen consumption (VO_2_max) or HRmax [41,42,43,44,45,46]. Exercise in hypoxic conditions can induce a localized or transient hypoxia, leading to the activation of cellular responses mediated by the hypoxia-inducible factor 1 alpha (HIF-1α). The activation of HIF-1α is crucial in the regulation of various cellular mechanisms, including glycolysis, angiogenesis, and adaptation to oxygen deprivation [47,48]. Studies have shown that hypoxic training stimulates erythropoietin production and promotes mitochondrial biogenesis, leading to improvements in oxygen delivery, oxidative phosphorylation, and overall metabolic efficiency [49,50]. These adaptive changes result in increased endurance, improved glucose metabolism, and fat loss [51]. Lately, the relationship between lung function and cardiometabolic conditions has also been highlighted. Previous studies have demonstrated that decreased lung function is associated with an increase in low-grade inflammation [52], diabetes risk, cardiovascular disorders, as well as metabolic syndrome [53,54].

The endocrine system controls the functions of other systems in the body, thereby regulating homeostasis. This is mediated by hormones released into circulation by various glands. Multiple organ dysfunction may be precipitated if there is any abnormality in the glandular secretion of hormones. The hormonal system integrates with the nervous system to achieve homeostasis. Consequently, neurological manifestations may be associated with endocrine dysfunction. The manifestation may vary from headache and myopathy to encephalopathy and coma. The dysfunction of the pituitary, pancreas, thyroid, and adrenal glands, causing diabetes mellitus, Addison’s disease, acromegaly, and Cushing’s syndrome, may show neurological manifestations [55]. Exercise interventions designed for individuals with neurodegenerative diseases are reported to increase strength, aerobic capacity, and balance [56,57]. Additionally, the latest research shows that exercise can avert or reduce disease progression in individuals with neurodegenerative diseases [58,59]. The recommendations from the American College of Sports Medicine (ACSM) also suggest that a minimum of 150 min/week of moderate exercise or 75 min of vigorous exercise or an optimal combination for best benefits of the geriatric population [60,61]. The objective of this review is to assess how age, body composition (BMI, obesity), lifestyle factors (smoking, alcohol use), and comorbidities (diabetes, hypertension, respiratory infections like COVID-19, neurodegenerative disease) modulate the physiological responses with exercise.

## 2. Methods

Relevant literature was retrieved from the electronic databases, including PubMed (https://pubmed.ncbi.nlm.nih.gov), Scopus (https://www.scopus.com), Google Scholar (https://scholar.google.com), and Web of Science (https://www.webofscience.com), from their inception to 17 June 2025. The article search was conducted using the following keywords: “exercise,” “physical activity,” “training”, “oxygen consumption,” “pulmonary ventilation,” “blood gases,” “chronic diseases,” “obesity,” “body mass index,” “diabetes,” “smoking,” “alcohol,” “COVID-19,” and “hypertension”. The search was conducted with or without a combination of “exercise,” “physical activity,” or “training” and other keywords. Randomized controlled trials (RCTs), clinical trials, and observational studies of human participants were selected and included in this review. Although we followed a strict search strategy, we did not restrict the number of articles to be included, and did not consider the risk of bias of the included studies. However, the quality of the studies was assessed based on their relevance to the topic, citation credibility, and quality of the journal. Our study is considered a narrative review due to the lack of strict inclusion or exclusion criteria and the risk of bias assessment results.

## 3. Variables Mitigating the Exercise Response

From the included articles, we identified multiple variables that influence the effects of exercise on oxygen consumption, pulmonary ventilation, and blood gases in an individual. Among these variables, age, sex, BMI, and lifestyle factors play an important role in modulating the physiological responses to exercise [62,63,64,65]. Aging, for instance, is associated with decreased muscle mass and altered metabolic efficiency, which can impair an individual’s ability to meet exercise-induced demands. These age-associated changes may reduce aerobic capacity and ventilatory efficiency [66]. Negative lifestyle factors, particularly smoking and alcohol consumption, decrease the functional ability of lungs and other organs, and thereby limit the metabolic efficiency of exercise [67,68,69,70,71]. In addition, pre-existing metabolic and chronic diseases, including obesity, diabetes, hypertension, CVDs, and neurodegenerative disorders, also influence the beneficial effects of physical activity [72,73,74]. In this article, we explored how these individual variables influence the oxygen consumption, pulmonary ventilation, and blood gas dynamics in individuals with chronic diseases.

## 4. Integration of Effects on Oxygen Consumption

Regular physical activity, including walking, cycling, running, and ergometer training, is widely recognized as a crucial part of lifestyle therapy for patients with CVD or coronary artery disease (CAD). These lifestyle interventions can decrease the risk of developing CVDs, CADs, heart failure, or other metabolic disorders [75,76]. Under normal conditions, the main limiting factor for VO_2_max is cardiorespiratory oxygen transport [77,78]. Quantified with incremental test protocols to exhaustion, VO_2_max cannot be regulated for long. Endurance exercise is reported to upregulate peroxisome proliferator-activated receptor gamma coactivator 1-alpha (PGC-1α) expression in skeletal muscle, leading to increased mitochondrial biogenesis and oxygen consumption [79,80,81]. As a master metabolic regulator, PGC-1α mediates critical exercise-induced adaptations, including fiber type transformation and angiogenesis, ultimately improving aerobic capacity and enhanced metabolic flexibility [80,82]. Higher and sustained VO_2_max levels signify greater efficiency in aerobic endurance exercises such as marathons, which reflects energy substrate availability, muscular energy expenditure, thermoregulation, and fatigue [83]. These physiological aspects of the respiratory system differ with gender [84,85]. In typical circumstances, comparable amounts of inspiratory effort for men and women produce the same level of diaphragm exhaustion, although women exert noticeably more effort for their body mass [86,87,88,89]. Variations in VO_2_max with exercise intervention among different people are presented in Table 1.

### 4.1. Age Factor

A study conducted on sedentary, overweight, or class-1 obesity adults in the age group of 40–65 years (middle-aged men and women) reported that exercising at a level of 19 km/week at 40 to 55% of peak VO_2_ is sufficient to increase aerobic fitness levels significantly above those of sedentary individuals, and simultaneously improve fitness and reduce cardiovascular risk; yet, higher intensities and amounts should be encouraged for additional benefit [90]. Similarly, another research group experimented with people by giving high-intensity interval training in the workplace. This study reported improvements in the markers of physical fitness (e.g., VO_2_max) and mental well-being (e.g., health-related quality of life and perceived stress). Future studies should aim to implement high-intensity exercise in the workplace in a variety of work settings (for example, manual work settings) with wider participation [91,99].

### 4.2. BMI

Overweight or obesity is associated with several health issues [100], and reduces respiratory capacity, including reduced lung (pulmonary) function like forced expiratory volume, forced vital capacity (FVC) and their ratios [101], and altered respiratory mechanics [101]. The possibility of exhibiting expiratory flow limitation, elevated respiratory resistance (setting ventilatory restriction), and a modified pattern of breathing throughout exercise (a reduced tidal volume resulting in high breathing frequency) becomes elevated in individuals with obesity due to deviations in respiratory mechanisms resulting in low lung volume respiration. Low lung volume respiration is defined as reduced functional residual capacity during rest and end-expiratory lung volume during exercise. Minute ventilation generated during exercise would be affected due to modified respiratory mechanics, which decreases ventilatory capacity [102] as well as ventilatory responses [103]. An inverse relationship was found to exist between increasing BMI and exercise ventilatory responses. This was high among women. Furthermore, maximum oxygen consumption increased with increasing BMI and obesity [104]. Likewise, a study adopted a corporate exercise intervention for 12 weeks and reported a significant decrease in cardiovascular risk factors, body weight and BMI, while there was an increase in VO_2_peak, and upper- and lower-body strength in adults. This indicates that the physiological fitness, muscle strength, and cardiovascular fitness of employees were enhanced, along with a decrease in employee productivity loss by 1.1% [92].

### 4.3. Smokers and Non-Smokers

According to the latest statistics from the WHO, approximately 22.3% of the global population use tobacco, with a significant gender difference: 36.7% of men and 7.8% of women are current tobacco users [105]. In addition, smoking has a negative impact on public health, with an estimated 422 billion US dollars in worldwide healthcare costs in 2012 being directly related to smoking [106]. Tobacco use or smoking remains a significant leading contributor to premature death and disability [107,108]. Therefore, among Asian males in their youth and middle years, active smoking lowers aerobic as well as anaerobic activity [109] through impaired perfusion of the pulmonary system and muscle, elevating blood lactate concentration, leading to reduced oxygen extraction due to the carbon monoxide effect [110,111]. Smoking also damages the vascular endothelium, increases free radical induction, affects blood flow in respiratory muscles, decreases respiratory muscle strength, and thus results in poor lung function [112,113]. Along with pulmonary pathologies, cigarette smoking can cause proinflammatory status, increase proteolysis, and inhibit protein synthesis, which leads to a loss of muscle mass [68,114].

### 4.4. Alcoholics and Non-Alcoholics

Emerging evidence suggests that regular exercise intervention can promote oxygen consumption, oxygen utilization, liver function, and cardiopulmonary function in adults with alcohol use disorder. Chronic alcohol consumption is reported to impair mitochondrial efficiency and exacerbate aging-induced cardiovascular capacity that eventually contributes to a diminishing aerobic capacity in adults [115,116]. Regular exercise, particularly moderate-intensity endurance exercise or resistance training, has been shown to reverse these adversities by improving mitochondrial biogenesis, endothelial function, and antioxidant defenses [70,117]. According to a study, a rigorous, planned, 10-week training program increases grip strength and cardiopulmonary endurance in the adult population with normal health. However, the concurrent daily consumption of moderate doses of ethanol or beer did not affect this. There were no appreciable gains in muscular activity parameters, but VO_2_ consumption was decreased in the non-alcoholic group. Therefore, moderate daily consumption of beer with meals, as in this study, does not appear to be a concern that it influences exercise variables like muscular and cardiopulmonary fitness following a HIIT program among the young healthy adult population. However, they also suggest a more elaborate study to confirm this relationship [94]. Despite these studies, we lack substantial evidence to relate alcohol and non-alcohol consumption with physical fitness and its connection with oxygen consumption [118]. Moreover, peak VO_2_ did not correlate with significant episodic drinking of alcohol. This result is consistent with randomized experiments showing that acutely high alcohol consumption has no effect on peak VO_2_ [71,119]. Prior studies have indicated that the beneficial effect of low-to-moderate intake may be counteracted by severe episodic drinking [120].

### 4.5. Diabetes

Exercise is a well-recognized intervention to provide substantial positive health benefits to patients both with and without diabetes. Nevertheless, it also poses complications to the endocrine system, which is responsible for preserving glucose homeostasis. Patients with diabetes, particularly those with T1D, have a significantly greater challenge because their primary complication is optimal glucose management [121]. Numerous studies have been conducted on exercise physiology, especially on the peripheral utilization of insulin [122,123,124]. The majority of research found that both the insulin-independent as well as insulin-dependent routes of glucose clearance are activated by physical activity, such as exercise, and that the insulin-dependent system continues to function after exercise. As a result, muscular sensitivity continues to develop [125]. This result was confirmed in vivo in a sophisticated investigation that used euglycemic treatment on three doses of insulin to assess insulin-dependent and insulin-independent glucose absorption in a T1D population [126]. Compared to patients without diabetes, patients with T1D are more likely to experience hypoglycemia spells during physical activity, particularly if counter-regulation is impaired [127].

An established walking program was found to enhance walking distance, predict aerobic ability, along cognitive abilities in older women with T2D. Additionally, walking enhances the consumption of oxygen in adults [128,129]. T2D is a distinct indicator for decreased peak VO_2_ in chronic heart failure patients with or without compromised left ventricular ejection fraction. In these cases, T2D may adversely affect the exercise outcomes; the extent of this effect is somewhat influenced by the systolic function of the left ventricle. In addition to lowering cardiovascular complications, diabetes prevention is crucial for maintaining exercise tolerance in the treatment of heart failure [95].

### 4.6. Hypertension

Human aging is characterized by increased aortic stiffness [130] and results in systolic hypertension by altering arterial compliance and increasing systolic wave reflections [131]. Aging, alongside hypertension and obesity, is a key risk factor for the progression of heart failure with preserved ejection fraction [132]. Hypertension is the most common symptom in end-stage renal disease patients undergoing hemodialysis, and its prevalence approaches 70–90% [133,134]. Although receiving antihypertensive treatment, 35% of patients failed to adequately control their hypertension, while exercise has been seen as an essential non-pharmacological strategy for controlling blood pressure through water and sodium regulation [135]. The combinational approach of using continuous aerobic physical training programs and epigenetic changes, such as DNA methylation diminishes the level of systemic blood pressure (hypertension) [136,137].

### 4.7. Parkinson’s Disease (PD)

Exercise training also increases the metabolism of the brain, and the beneficial effects of exercise depend on the crosstalk between nervous tissue and muscles [79]. More than skeletal muscle, the brain is more vulnerable to an oxygen drop [138]. The increase in cerebral blood flow during exercise maintains brain oxygenation [139]. Physically fit older people had fMRI (functional brain MRI) evidence of significantly improved cortical connectivity and activation during cognitive tasks compared to their unfit counterparts, as assessed by peak VO_2_ during exercise [140,141]. Data on the efficiency of progressive resistance training in the recovery of people with PD are rare. Recent systematic reviews show that progressive resistance training has a positive effect on muscle strength, mobility, endurance, lean body mass and performance on functional tasks, and non-motor symptoms [142,143], and it may be effective in increasing walking ability in PD [144] but maximal oxygen consumption was below the level of minimal clinical significance in one of the studies [145]. A study by Shulman and group tested three different types of exercises (higher-intensity treadmill training, lower-intensity treadmill training, stretching, and resistance training), and reported that both low- and high-intensity treadmill exercises improved maximum oxygen consumption more than stretching and resistance training. Thus, a combination of treadmill and resistance exercises has proven beneficial effects in PD, which also requires further investigation [145]. Another study reported similar results with aerobic walking in a community setting, and found improved aerobic fitness, fatigue, executive control, mood, and motor abilities, as well as well-being, among mild-to-moderate PD cases, along with improved maximum oxygen consumption [145,146]. The effect of maximal effort graded exercise test also found higher oxygen consumption in patients with mild to moderate PD [147]. In the clinical context, when prescribing exercise training (either aerobic or resistance) to patients with PD, the training protocol should be tailored based on the patient’s physical fitness, acceptance, and disease status.

### 4.8. COVID-19

In one study, patients who recovered from COVID-19 performed cardiopulmonary exercise testing for 3 or 5 months, and it was found that their peak oxygen consumption had reduced significantly [148]. Mild exercise is advised to increase cardiovascular health and physical fitness, but for added benefits, more vigorous levels and quantities should be encouraged. For class 1 obese patients, at least 19 km/week needs to be covered by exercising, which enhances their aerobic fitness and maximum oxygen consumption [149]. But for those with a high BMI, oxygen consumption is also higher than the baseline value; hence, exercise helps in the reduction of VO_2_max [150]. Smoking reduces the extraction of oxygen due to the effect of carbon monoxide in young and middle-aged Asian males. Hence, the authors suggest that while doing some form of exercise will not make smokers healthy it can ameliorate their health condition to some extent [151]. The consumption of beer or low concentrations of alcohol, along with structured exercise, does not affect respiratory fitness. However, this persists only when they are consumed at a healthy level (12 fluid ounces of beer or 5 fluid ounces of wine per day, or two in a day) [94]. In patients with diabetes (comorbid with a heart failure condition), exercise like a structured walking program helps in the clearance of glucose from the body in both insulin-dependent (T1D) as well as insulin-independent (T2D) conditions [152]. Aging, comorbid with hypertension and obesity, is the crucial factor in the development of heart failure. In neurodegenerative disorders, exercise improves the ability to perform cognitive tasks [153].

## 5. Integration of Effects on Pulmonary Ventilation and Blood Gases

### 5.1. Pulmonary Ventilation

The maximal inspiratory pressure (MIP) and maximal expiratory pressure (MEP) are used to measure the strength of the muscles involved in respiration. The MEP and MIP are derived from total lung capacity (TLC) and residual volume, respectively [154]. The method used to assess the chest wall volume included optoelectronic plethysmography. Based on Gauss’ theorem, the system, which comprises eight cameras that can record the information from eighty-nine markers, determines the alterations in the volume of the chest wall overall and in each compartment using the coordinates of the markers [155]. The program makes it possible to create a three-dimensional replica of the chest wall following the registration procedure [156]. The methodology for the acquisition included two minutes of silent breathing before the technique was applied, two minutes of quiet breathing after the technique was applied, and three acquisition times of two minutes each (instantaneously after the technique; 15 min and 30 min post-technique). Total chest wall volume and its compartmental volumes—pulmonary rib cage volume (rcp), abdominal rib cage volume (rca), and abdomen volume (ab)—were chosen for analysis, and the variables were evaluated in the second minute after the breathing pattern returned to normal [157].

Exercise also improved minute ventilation (pulmonary ventilation) in pregnant women (gestational age < 13 weeks) in 29–37 years the age group when treated with submaximal cardiopulmonary exercise testing [158], and also in adults following a spinal cord injury, when treated with exoskeleton-assisted walking [159]. Singing has always been a part of every culture, whether ancient or modern [160]. Less research has been carried out on the cardiopulmonary requirements of singing as well as the benefits it provides as an exercise and daily physical activity adjunct. Researchers found that singing while standing caused strong physiological processes that were comparable to moderate exercise. This study also discovered that during singing and related activities, there was an increase in minute ventilation along with breath volume. When taking into account the possibility of the transmission of respiratory illnesses like the SARS-CoV-2 infection, the benefits seem clear. These findings imply that the positive effects of singing on health and happiness may stem, at least in part, from physiological processes [161].

Cardiac rehabilitation improved the peak respiratory exchange ratio (VE/VCO_2_) in heart failure patients with a low BMI [162]. Cardiac rehabilitation also improved the pulmonary function of patients with a high body weight and obesity [14]. In patients with obesity, there is reduced exercise performance, but obesity alone is not responsible for this reduction. Obesity does not account for the decreased exercise performance seen in obese patients. Exercise is severely limited by unilateral diaphragm paralysis and obesity, which reduces peak oxygen consumption and the peak work rate and makes breathing difficult. It is important to advise patients who have unilateral diaphragm paralysis and dyspnea not to put on weight [163]. When pulmonary rehabilitation is implemented in COPD patients, exercise time is improved instead of exercise tolerance, which saves oxygen consumption and lowers the need for ventilation without increasing cardiac loads, which is followed by a decrease in exertional dyspnea. Furthermore, pulmonary rehabilitation responses may be predicted using the time-slope and BMI [164].

Despite the extent of pollution in the air at residential regions throughout Western Europe, physical exercise shows a significant influence on adult pulmonary function as well as health in active smokers; however, the changes in non-smokers in high-pollution locations are yet unknown. However, in never-smokers residing in areas with significant air pollution, there might be some reduction in such (physical activity) benefits. If this were the case, laws regulating air pollution would guarantee that a program promoting physical exercise would have the greatest possible impact [165]. To avoid COPD in smokers as well as in non-smokers, it is very important to avoid secondhand smoke exposure outside the home, as it decreases the forced expiratory volume in these people [166]. Deep breathing exercise techniques were found to be useful in healthy smokers (smoking history of 5 years) for improving lung function and delaying the development of chronic obstructive lung complications [167]. There is mounting evidence that regular exercise enhances lung function; these correlations seem to be higher in active smokers [69,168].

Several lines of evidence indicate that alcohol exposure during fetal life is associated with significant disorders in central respiratory areas, disrupting brain control over breathing and causing abnormal breathing patterns, despite the paucity of experimental research. These disruptions had an impact on the basal conduction characteristics as well as the nervous system’s ability to long-term as well as short-term acclimatization to reduced oxygen levels. Significantly, and as a result of the latter findings, exposure to ethanol during this particular developmental stage might represent an important predictor of sudden infant death syndrome. This study was conducted on rat pups and bullfrog tadpoles [169].

It has been noted that people living with T1D and T2D have decreased lung function [170,171]. The FVC, forced expiratory volume in 1 s (FEV1), and the corresponding ratio have been the main cross-sectional connections made between diabetes conditions and respiration metrics that have demonstrated this physiological compromise [172]. Impaired FEV1% (estimated to be 2.8% with T1D and 4.9% with T2D) and FVC% (predicted to be 3.8%t with T1D and 6.7% with T2D) are two ways that diabetes-related impaired lung functions are exhibited [172]. Diabetes is not a strong predictor of pulmonary function decline over time [173,174,175], which suggests that, unlike other consequences of diabetes with microvascular pathogenesis, including nephropathy, retinopathy, and peripheral neuropathy, the respiratory problems in diabetes are non-progressive. In certain patients, combined pancreas and kidney transplantation for the reversal of diabetes has been demonstrated to improve FEV1 and FVC decline [176]. Further studies are required to comprehend the impact of the physiological processes relating to decreased lung function and diabetes to develop adequate therapies targeting COPD as well as diabetes [177].

The incremental shuttle walking test is a straightforward method for evaluating the peak exercise capacity in patients with pulmonary arterial hypertension (PAH). It is also susceptible to treatments and outcome indicators, and is free of ceiling effects [178]. The right ventricular contractile reserve declines due to hypoxia-induced vasoconstriction among healthy controls, and chronic PAH cases exhibit marked contractile reserve depletion regardless of a healthy ejection fraction while resting, as determined by noninvasive cardiac imaging throughout submaximal exercise. Right ventricular dysfunction may be revealed by exercise imaging before signs of resting responses to PAH become apparent [179]. According to one investigation, individuals with COPD may have attenuated angiogenesis since they demonstrated less training-induced blood pressure modification linked to a shift in muscle capillarization throughout exercise [180].

In neurodegenerative CNS disorders, a decreased maximal inspiratory pressure and maximal expiratory pressure, along with a restrictive pattern of spirometry, are typical features of pulmonary function. Although there are minor effects on pulmonary function in the acute phase, vital capacity as well as FVC are chronically significantly diminished when there is considerable muscular weakening [181,182]. Studies from inspiratory muscle training revealed that patients suffering from multiple sclerosis and Parkinson’s disease showed significantly altered dyspnea patterns, strength of diaphragm, pulmonary volumes, pulmonary capacities, and endurance, when the training intervention consisted of at least 30 inspiratory maneuvers each day, 6 days a week, for 10 weeks, at 30% of the maximal inspiratory pressure, with progressively increased load [183]. When Parkinson’s disease progresses, patients typically manifest with shorter lung capacities along with reduced respiratory volumes, which increases the risk of pneumonia due to aspiration, one of the prevalent causes of mortality [184]. Previous research has demonstrated a reduced lung volume and capacity in Parkinson’s disease cases, most likely as a result of decreased respiratory muscle coordination and strength, as well as reduced chest compliance brought on by musculoskeletal restrictions in the rib cage [181,185].

In an investigation, individuals who have survived COVID-19 pneumonia should undergo a thorough respiratory screening and rehabilitation at the peak acute phase, and finally be directed to specific care to monitor and treat serious complications over the follow-up period [186]. Pulmonary fibrosis in geriatric cases has better outcomes in cardiorespiratory strength and quality of life through respiratory rehabilitation, including exercises. The six-minute walking distance (6MWD) significantly improved in younger cases, and cases with a moderate exercise intensity or aerobic resistance exercises after excluding factors like BMI variations and exercise duration. Regarding pulmonary function, an improvement was seen with FVC, while the diffusing capacity of the lungs for carbon monoxide (DLCO) and total lung capacity (TLC) were unaffected [187]. Patients who have heart failure and a low BMI can experience cardiac rehabilitation, which improves the respiratory exchange ratio (VE/VCO_2_). Also, patients with overweight and obesity have better pulmonary function when they perform COR. To regulate lung function in smokers and to avoid the development of lung-related complications, deep breathing has been proven effective. Not many studies have been undertaken to estimate the changes in pulmonary function during exercise in alcoholics and non-alcoholics, as it mostly affects the liver and rarely affects the lungs. Hence, future studies can help in understanding this complex relationship between alcoholics and their lungs during exercise. Diabetes reduces pulmonary function, and exercise might help in maintaining these functions. An incremental shuttle walking test enhances pulmonary arterial hypertension by reducing right ventricular contractile reserve. Even significant improvement has been seen in neurodegenerative disorders like PD and multiple sclerosis.

### 5.2. Blood Gas Dynamics

Arterial oxygen saturation at assumed alveolar oxygen tension can be improved by increasing hemoglobin’s affinity to oxygen, which shifts the oxygen–hemoglobin dissociation curve to the left [188,189], thereby reducing symptoms of hypoxia. However, increasing hemoglobin’s affinity to oxygen at the assumed oxygen delivery rate decreases oxygen’s perfusion to tissues. These kinds of trials of modifying hemoglobin’s affinity to oxygen had variable outcomes [190,191,192].

Age-related changes in the tolerance of exercise and their causes in healthy persons were examined in another investigation. The main conclusion indicates that arteriovenous oxygen difference, which is a measure of skeletal muscle’s diminished capacity for oxygen uptake, caused a decreased tolerance to exercise in the geriatric population. Additional research indicates that cardiac contractility and output are maintained during aging. In healthy elderly individuals, there is a considerable decrease in the maximum heart rate; nevertheless, due to reflex activity, stroke volume rises dramatically, enabling an optimum cardiac output comparable to younger individuals [193]. Elderly persons have lower heart rates along with a low VO_2_peak [194]. Arteriovenous oxygen variations and cardiac output are the two peripheral and central physiologic variables that contribute to a shorter VO_2_peak [195]. The geriatric group showed a greater correlation between arteriovenous oxygen variation and VO_2_peak in comparison to the younger group, indicating that the primary factor influencing tolerance to exercise in older adults is the skeletal muscles’ capacity to uptake supplied oxygen [196]. Age-related alterations in the circulatory system are exacerbated, peripheral O_2_ utilization is significantly reduced, and physiological activity deteriorates as a result of a reduction in exercise intensity and quantity combined with increasing sedentary habits [197,198]. On the other hand, enhanced peripheral mechanisms, such as capillary circulation and musculoskeletal activity, mitigate the age-related decline in arteriovenous oxygen variation among older adults during increased exercise in both quantity and intensity [74]. The decreased sympathetic activity in aging is the primary contributor to the decreased cardiovascular activity and declining cardiac output [199].

A considerable increase in stroke volume during functional stress, which compensates for the reduced heart rate in older people, indicates that the maximum cardiac output is unaltered by age in many investigations with subjects ranging in age from 20 to 80 years [194,200]. Through the Frank–Starling mechanism and a marked increase in end-diastolic volume, stroke volume is increased [194,201]. Stroke volume was formerly believed to plateau at about half of VO_2_peak among subjects with normal health; however, recent study data have shown that stroke volume expands gradually during maximum activity testing, especially in those who are trained [194,200]. Heart rate variations have been linked to an age-associated decrease in VO_2_peak, which has 5% incidence rate each decade and is independent of sex or lifestyle [62]. It would be interesting to see if maximum heart rate influences cardiorespiratory activity, as there is an association between decreasing VO_2_peak and maximum heart rate [62].

In the context of body weight, for patients with obesity who have metabolic syndrome, exercising in hypoxic settings may partly enhance the myocellular insulin sensitivity that can be attributed to exercise alone. Simultaneous vascular endothelial growth factor (VEGF) changes could indicate a pathogenic cause [202]. A study showed that among healthy individuals, increasing hemoglobin’s oxygen affinity enhances arterial oxygen saturation throughout hypoxia with no impact on VO_2_ or circulatory responses during optimal exercise. Further, minimal hyper-ventilatory responses showed an increase in blood oxygen saturation (SaO_2_), causing a decreased arterial carbon dioxide pressure (PaCO_2_) and increased arterial oxygen pressure (PaO_2_) [203]. Despite hypoxia-induced spikes during physiological strain, eight sensibly guided interval-walk activities over two weeks of training among obese people produced identical treadmill velocity along with perceptual reactions at hypoxia and normoxia. Blood pressure, weight reduction, and functional performance did not significantly differ between the two training conditions, despite the fact that both strategies enhanced exercise-related signals. For people who are obese, hypoxia is unlikely to alter certain cardiometabolic complications or enhance exercise tolerance, at least not after a brief training session [204].

Acute exercise to exhaustion leads to a significant decrease in endothelin-1 (ET-1) levels in young, active, healthy individuals. Despite being young, active, and otherwise healthy, chronic smokers were not able to achieve the same decrease in ET-1 as non-smokers, probably due to an underlying shortage of bioavailable nitric oxide and/or impaired respiratory clearance [63]. By releasing a variety of vasoactive chemicals, the vascular endothelium contributes dynamically to the ongoing regulation of the tone of the vascular system. In this role, ET-1 is essential since it is the strongest vasoconstrictor [205]. Hypoxia damage to tissues, or anxiety, increases the generation of ET-1, which then quickly upregulates the activity of ET_A_ along with ET_B_ receptors to exert its physiological responses. In particular, vascular smooth muscle cells (VSMCs) comprising ET_A_ as well as ET_B_ receptors regulate the vasoconstrictor effects of ET-1 [206].

One-third of smokers did not exhibit impaired lung function or chronic manifestations. However, very-light smokers exhibited inflammatory, cytological, and impaired functional changes [207]. Specifically, alveolar–capillary membrane conductance (ACMC) was affected. Asymptomatic smokers exhibited a minimal exercise-induced rise in DLCO, which reveals initial pulmonary damage due to smoking that is not identified by traditional pulmonary function diagnostics [208].

Short-term exposure to air pollutants such as PM 2.5, NO_2_, and O_3_ is associated with increased liver enzyme levels in older people. These adverse effects can be reduced by exercising regularly and abstinence from alcohol [209].

It is well known that T1D damages peripheral tissues, but very little is known about its effects on the pulmonary system. In pulmonary ventilation, the diffusion capacity, and DLCO is determined by ACMC and pulmonary–capillary blood volume (PCBV). These two are affected in T1D due to capillary basal lamina thickening and the increased permeability of the endothelium [210,211]. Furthermore, in healthy T1D patients, there was a decrease in DLCO during exercise affecting ACMC. However, there was no significant worsening of DLCO in most T1D patients who had good glycemic control during a 5-year follow-up period. Vigorous exercises had detrimental effects on DLCO in chronic diabetics who had lower DLCO [212]. In particular, low DLCO affected ACMC more than PCBV, which confirms the pathology of the alveolar–capillary membrane in T1D [210].

Patients with chronic pulmonary hypertension (PH) are becoming more and more concerned about the possible negative health implications of flight travel or being at a high altitude. An initial randomized, placebo-controlled study demonstrates that, though there is significant inter-individual variability, PH patients also have a decreased capacity for exercise in hypoxic settings, similar to normal subjects and COPD patients. As dyspnea, cardiac output, pulmonary circulation after exercise, and muscle deoxygenation at the time of exercise did not change, the complications of exercise under hypoxia were likely due to increased ventilation and a reduced level of oxygen, as confirmed by decreased PaCO_2_ after exercise [213]. The six-minute walking treadmill (6MWT) test is the principal way to test the six-minute walking distance (6MWD). A decreased 6MWD due to a ceased 6MWT test with the constraints of desaturation would be an inevitable repeating outcome when restarting the 6MWT after peripheral capillary oxygen saturation (SpO_2_) recovery. This decreased 6MWD can impact some patients in terms of risk stratification as well as treatment access, like in the USA, this will affect lung allocation scores, indicating the need for a priority lung transplantation. The outcome of the 6MWT test would be desaturation for lung diseases like PH and interstitial lung disease compared to other cardiorespiratory diagnoses. The 6MWD is critical in clinical decision-making, and cessation of the test before completion would impact the diagnostic outcomes in such patients [214].

Patients with PD may have improvements in their maximal and submaximal cardiorespiratory performance following aerobic exercise. Among this exercise group, the factors that were best indicators of optimized VO_2_peak were a reduced initial VO_2_peak, greater exercise pace, and younger age. The gains in aerobic ability that were observed emerged following a rather brief aerobic cycling exercise [215]. Table 2 gives an overview of the effect of exercise on pulmonary ventilation and blood gas. Very few studies (at least in the human model) have examined the effect of exercise on arterial blood gas in neurodegenerative disorders, or even if found, no significance was observed [216]. Exercise helps in decreasing age-related arteriovenous O_2_ differences via microvascular circulation and the functioning of skeletal muscles. Smoking lowers the gas exchange efficiency in the muscles and lungs, elevating blood lactate concentration, damaging the vascular endothelium, increasing free radical production, affecting blood flow in the respiratory muscles, and causing poor lung function, which can be cured only by a suitable exercise regimen. In patients with diabetes, the DLCO is high, but during physical activity, it is lowered, which is attributed to low alveolar–capillary membrane conductance. Patients with PH have a reduced exercise capacity, and the 6 min walk test (6MWT) can help in enhancing the exercise capacity. The effect of arterial blood gases on neurodegenerative disorders needs to be explored further to study the effect of exercise.

### 5.3. Limitations of Current Evidence

This narrative review has several limitations, which should be further explored. First, the safety and effectiveness of HIIT in patients with severe hypertension and heart failure remain unclear. Although HIIT may enhance VO_2_peak more effectively than MICT, its safety in patients with reduced ejection fraction (HFrEF) remains uncertain [18]. Similarly, data regarding HIIT in heart failure with compromised ejection fraction (HFpEF) are limited and inconclusive [222]. Second, determining the optimal exercise training dosing is also very difficult due to the variation in HIIT protocols adopted across different populations and BMI categories [17,223]. Third, the study populations (e.g., diabetics, hypertensive patients, smokers) frequently presented with multiple comorbidities, making it impossible to establish pure cohort comparisons or definitive baseline values for each subgroup. Fourth, the methodological heterogeneity across studies resulted in inconsistent reporting of outcomes, while some investigations provided statistically significant data for HIIT, MICT, or other exercise types, others did not, introducing a risk of selection bias into our article [224]. In addition, data collection related to oxygen consumption, pulmonary function, and blood gas parameters was constrained by considerable variations in measured outcomes in the studies and incomplete reporting of selected outcomes. Consequently, our findings represent only the most consistently reported variables, which may provide an incomplete picture of exercise effects and highlight the need for individualized approaches and population-specific guidelines.

## 6. Conclusions

**Figure 1:**
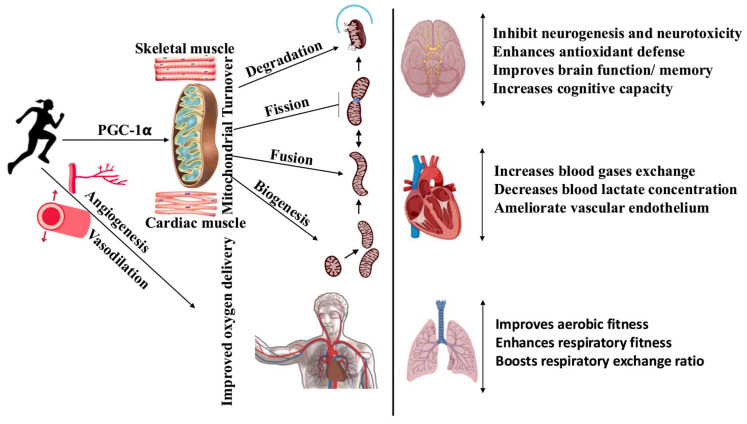
Effect of exercise on physical fitness and functioning of the brain, heart, and lungs. The arrowhead is a promotion, and the flathead is an inhibition.

In this review, we comprehensively discussed the physiological effects of exercise on oxygen consumption, pulmonary ventilation, and blood gases. Evidence demonstrates that exercise enhances oxygen consumption, pulmonary ventilation, and blood gas exchange by increasing minute ventilation, improving cardiopulmonary function, and optimizing venous CO_2_ levels. The implementation of brief exercise programs in the workplace could be an effective strategy for busy individuals to maintain a healthy lifestyle, promote mental health, and decrease the risk of metabolic diseases, without disrupting work productivity. Regardless of type or intensity, physical exercise influences multiple systems, including the musculoskeletal, circulatory, respiratory, and neuroendocrine systems, through paracrine mediators like nitric oxide and reactive oxygen species (Figure 1). Studies on children with Kawasaki disease and COVID-19 survivors suggest that exercise may improve ventilatory responses and pulmonary function, thereby potentially enhancing quality of life. However, exercise regimens, either aerobic or resistance, should be tailored carefully, especially for individuals with underlying health conditions (CVDs or neurodegenerative disorders), and medical consultation is recommended before starting any regimen.

## Figures and Tables

**Table 1:** Integration effects of variables on oxygen consumption in response to exercise.

Variables	Sample Size (*n*)	Age in Years	Intervention	VO_2_max (mL/kg/min)	Study Reference
Age	37	52.2 ± 7.1	Control	27.4 ± 5.7	[90]
	25	53.7 ± 5.2	Low amount/moderate intensity	29.6 ± 6.9	
	36	52.0 ± 6.9	Low amount/high intensity	32.4 ± 6.4	
	35	50.9 ± 5.4	High amount/high intensity	34.6 ± 6.1	
Workplace	24	46 ± 12	Control	36.6 ± 9.0	[91]
	30	46 ± 9	Brief exercise intervention program	37.7 ± 7.5	
Body Mass Index	251	>18	Corporate exercise intervention program	43.1 ± 16.5 *	[92]
Smokers	6	23.0 ± 1.4	Taekwondo athletes	58.4 ± 10.0	[93]
Non-smokers	9	22.4 ± 1.3	Taekwondo athletes	62.23 ± 6.1	
Alcoholic	6	25.2 ± 5.5	High-intensity interval training	44 ± 10	[94]
Non-alcoholic	8	19.9 ± 2.3	High-intensity interval training	41 ± 8	
Diabetes	34	61 ± 12	Cardiopulmonary Exercise Testing	15.16 ± 3.82	[95]
Non-diabetes	97	54 ± 17	Cardiopulmonary exercise testing	17.46 ± 5.22	
Hypertensive	53	47 ±14	Cardiopulmonary exercise testing	13.4 ± 3.6	[96]
Parkinson’s Disease	23	66.1 ± 9.73	Higher-intensity treadmill exercise	22.39 ± 0.9	[97]
	22	65.8 ± 11.5	Lower-intensity treadmill exercise	25.11 ± 1.4	
	22	65.3 ± 11.3	Stretching and resistance exercises	22.89 ± 1	
COVID-19	18	50 ± 9	Mild–moderate disease	22.1 ± 6.3	[98]
	18	58 ± 13	Severe disease	18.4 ± 5.0	
	39	59 ± 11	Critical disease	19.8 ± 5.1	
Kawasaki disease (Children)	7	9.7 ± 0.5	At rest	6.5 ± 0.4	[65]
			Treadmill exercise progressive test	46.1 ± 1.7	
Kawasaki disease (Young adults)	6	18.0 ± 1	At rest	4.5 ± 0.3	
			Treadmill exercise progressive test	45.5 ± 1.3	

Values are mean ± standard deviation unless otherwise indicated; * median (IQR) values; VO_2_max reported in milliliters of oxygen per kilogram of body weight per minute (mL/kg/min).

**Table 2:** Integration effects of variables on pulmonary ventilation and blood gas dynamics in response to exercise.

Variables	Pulmonary Ventilations	Blood Gas Dynamics
Age Factor	During exercise testing in pregnant women, minute ventilation increased from 12 L/min to 28 L/min during 1 min of rest [158].	Maximal arterial–venous oxygen difference (A-VO_2_ diff) was higher in exercise-trained individuals (19.8 ± 4.0 vs. 17.3 ± 3.7 mL/dL; *p* = 0.03) [74].
		The A-VO_2_ difference at rest was 5.4 ± 1.7 in young people and 4.3 ± 1.6 in older people; during maximal exercise, it increased to 15.4 ± 2.6 in young people and 10.1 ± 1.8 in older people, indicating that the A-VO_2_ difference was greater in young people [194].
Body Mass Index	After cardiac rehabilitation, the %∆peak VO_2_ per 1mL/min increase was significantly higher in patients with lower BMI (17.1 ± 2.8% vs. 7.8 ± 1.5%; *p* < 0.001) [162].	SaO_2_ did not change at rest in normoxia, but increased during exercise on day 15 (96.6 ± 0.3% vs. 95.2 ± 0.4%, *p* < 0.05). In hypoxia, SaO_2_ increased at rest (90.9 ± 1.8% vs. 82.9 ± 3.4%, *p* < 0.05) and during exercise (84.8 ± 2.7% vs. 73.6 ± 2.5%, *p* < 0.01) on day 15. PaO_2_ increased and PaCO_2_ decreased after treatment [203].
		SaO_2_ levels during interval walking in individuals with obesity were 83 ± 1% in hypoxia and 96 ± 1% in normoxia, but this difference was not statistically significant (*p* > 0.05) [204].
Smokers	In healthy smokers, deep breathing exercises caused a significant change in FVC, inspiratory capacity, tidal volume, expiratory reserve volume, and FEV1 (*p* < 0.05) [167].	Smoking significantly increases the difference in O_2_ pressure differences (p(A–a′)O_2_) and arterial ((a′)–end-tidal (et)) carbon dioxide (CO_2_) pressure differences (p(a′–et)CO_2_) during rest and peak exercise, while dead space/tidal volume ratios (VD/VT) increase significantly only during exercise [64].
Non-Smokers	After maximal treadmill exercise, endothelin-1 levels were significantly reduced in non-smokers (*p* < 0.001). Chronic smokers showed fewer exercise-related changes in tidal volume (*p* = 0.050), fraction of expired CO_2_ (*p* = 0.021), oxygen consumption (*p* = 0.005), CO_2_ elimination (*p* = 0.004), and peak expiratory flow (*p* = 0.003) [63].	
Alcoholic	A 90-day running program in a patient with alcohol addiction increased his running time from 6 to 45 min, with a VO_2_max increase from 24.2 to 30.1 mL/kg/min) [217].	Exercise attenuated the increased liver enzyme levels in older people exposed to air pollutants [209].
Diabetes	In an incremental exercise test, patients with type 1 diabetes showed lower aerobic capacity than healthy controls, with reduced VO_2_ (41.57 ± 7.68 vs. 51.12 ± 9.94 mL/kg/min), lower VE (76.39 ± 19.93 vs. 96.90 ± 25.72 mL/kg/min), and shorter time to exhaustion (8.75 ± 1.60 vs. 10.82 ± 1.44 min) [218].	During hyperoxic exercise, the oxygen-binding pressure (pO_2_) in the blood of patients with type 2 diabetes increased significantly (*p* < 0.05), but there was no change in arterial pCO_2_ [219].
Hypertensive	In individuals with hypertension, the distance covered in the incremental shuttle walk test was significantly associated with hemodynamic parameters at baseline (*p* < 0.001). Further, both at baseline (AUC = 0.655; *p* = 0.004) and at 1 year after initiation of treatment, distance (AUC = 0.737; *p* < 0.001) could able to predict mortality [178].	In COPD outpatients, at peak exercise, PaO_2_ (<8.5 kPa) better predicted mean pulmonary artery pressure and pulmonary hypertension than PaO_2_ at rest (<9.5 kPa) [220].
Parkinson’s Disease	Respiratory muscle training improved respiratory volumes and lung capacities in patients with Parkinson’s disease and multiple sclerosis [183].	Patients with Parkinson’s disease show poor exercise tolerance and respiratory muscle weakness and pulmonary function; exercise training improved FVC [221].
COVID-19	Exercise training in elderly patients with pulmonary fibrosis significantly improved 6 min walk distance by 34.04 m, peak VO_2_ by 1.13 mL/kg/min, and predicted FVC by 3.94% (d = 0.42, *p* = 0.01) [187].	

Abbreviations: A-VO_2_; arterial-venous oxygen consumption, BMI; body mass index, SaO_2_; blood oxygen saturation, PaCO_2_; arterial carbon dioxide pressure, PaO_2_; arterial oxygen pressure, FVC; forced vital capacity, FEV1; forced expiratory volume in 1 s; VD/VT; dead spaces to tidal volume ratio, VE; peak pulmonary ventilation, pO_2_; partial pressure of oxygen. pCO_2_; partial pressure of carbon dioxide.

## Data Availability

The data used in this article are available with the corresponding authors based on request.
